# Searching for health information on the Internet - Results from the KomPaS study

**DOI:** 10.25646/7147

**Published:** 2021-06-16

**Authors:** Kerstin Horch

**Affiliations:** Robert Koch Institute, Berlin, Department of Epidemiology and Health Monitoring

**Keywords:** SEARCH FOR HEALTH INFORMATION, INTERNET, SEX, SOCIAL STATUS, KOMPAS STUDY

## Abstract

Searching for information about health is a key component of health behaviour. It is important because information generally has a significant impact on the outcome of decision-making processes, and this also applies to informed decisions about health-related issues. Representative results from the study ‘KomPaS: survey on communication and patient-safety’, which was conducted by the Robert Koch Institute, demonstrate that the Internet is the most popular choice for women and men who use media to search for health information. However, the results also demonstrate statistically significant differences by sex and socioeconomic status. People in the low socioeconomic status group search less frequently for health information on the Internet than people in the medium and high status groups. Furthermore, women up to the age of 65 use the Internet to search for information about health more frequently than men of the same age do. These differences reverse from the age of 65 onwards.

## Introduction

The Act to Improve the Rights of Patients affords patients and the wider German population the right to have comprehensive health information provided in a manner in which they can understand. Better levels of information and transparency are essential for strengthening the role of health-system users, both to choosing health care services and sharing the responsibility for maintaining and regaining health [1]. These points are particularly relevant in light of the fact that information generally has a significant impact on the outcome of decision-making processes [2], and this also applies to informed decisions about health-related issues. From a public health point of view, the search, contextualisation, evaluation and implementation of health information by users as part of their health-related practices are essential aspects of health literacy. Therefore, people need to be provided with suitable information but also to be in a position to make decisions that meet their objective (evidence-based) and subjective (preference-based) needs for health care services. Since 2001, reports by the Advisory Council on the Assessment of Developments in the Health Care Sector have repeatedly emphasised this relation and the relevance of this topic in attempts to reduce over, under and incorrect provision of care [3].

In this context, the development and establishment of standards, prerequisites and structures that guarantee quality-assured, evidence- and needs-based health information are becoming increasingly important. Examples include the website informedhealth.org, and the German National Health Portal.

Trend analyses demonstrate that the Internet is becoming an increasingly important source of health information [4–6]. While the Robert Koch Institute’s (RKI) German Health Update (GEDA) 2009 [7] found that just 36.2% of the German population used the Internet to find health information, the study ‘KomPaS: survey on communication and patient-safety’, also conducted by the RKI, found that the figure had risen to 68.9% by 2017. At the same time, a wider range of health-related content became available on the internet [2]. Online health information-seeking behaviour is now a widespread health-related form of behaviour and is viewed as essential to empowerment and health literacy. Quality-assured, user-friendly, gender-appropriate informational strategies are required to improve the potential of the Internet for health literacy and patient empowerment [8–11].

**KomPaS study**

KomPaS: survey on communication and patient-safety

**Data holder:** Robert Koch Institute

**Objectives:** Describe informational needs, health literacy, patient safety, informed decision-making and physician’s counselling from the population’s point of view as part of patients’ information, decision-making and communication-related behaviour and the doctor-patient relationship.

**Survey method:** Computer-assisted telephone interview survey

**Study design:** Cross-sectional study

**Population:** German-speaking resident population in private households in Germany aged 18 or over

**Sampling:** Telephone sample comprising 60% landline and 40% mobile phone numbers

**Survey period:** May to September 2017

**Response rate:** 17.2%

**Sample size:** 5,053 participants

In summary, the results from the analysis of German and English-language overviews and a comprehensive literature review demonstrate the need for regular, standardised representative surveys. These surveys should assess the population’s health information-seeking behaviour and take into account traditional and digital information channels as well as key determinants such as age, gender and socioeconomic status. This is the only way to observe changes in information-seeking behaviour across different forms of media. Moreover, studying these changes is essential if we are to ensure that health information is drawn up appropriately, and that it is properly targeted and tailored to people’s particular needs.

## Indicator

The KomPaS study (Info box) was conducted within the RKI’s health monitoring framework. Data on searching for health information was gathered using questions about how often seven types of media (radio/television, the Internet, health apps, booklets or brochures from health insurers, booklets or brochures from chemists, health topics in other magazines or newspapers, and medical hotlines provided by health insurers) were used to search for health information. The respondents could choose from the following response categories: ‘often’, ‘sometimes’, ‘rarely’ and ‘never’. The respondents also had the option to use a free text field to report any other sources they had used to gain health information (such as information from doctors, relatives and friends).

This paper presents the results of the search for health information by the population in Germany. It concentrates on the types of media described above that the population ‘often’ uses to search for health information. The results are presented as prevalences and are listed separately for women and men. The analysis clearly demonstrates that the Internet is the most frequently reported source of health information in the ‘often used’ category. As such, this article focuses on the ‘search for health information on the Internet’ indicator and provides results (prevalences) for people who stated that they ‘often used’ the Internet to search for health information. Prevalences are stratified by sex, age group and socioeconomic status, and based on 95% confidence intervals. Statistical methods were used to test for significant differences between these groups. Statistically significant differences between women and men and/or the other (socioeconomic) groups under consideration are indicated. A statistically significant difference between groups is assumed when the corresponding p-value is lower than 0.05. The analyses were carried out descriptively using the survey procedures available from STATA SE 15.1 [12].

The analyses are based on data from a total of 5,053 participants aged 18 or over (56.7% women, 43.3% men). In order to ensure that the results are representative of the German resident population, the calculations used a weighting factor to correct for deviations within the sample from the actual population structure (as of 31 December 2016).

## Results and discussion

The data from the KomPaS study demonstrate that people are very interested in health-related topics. Just 1.9% of the participants (1.4% of women, 2.5% of men) indicated that they do not use any of the listed sources of information. Moreover, those who do use media use an average of four different types to search for health information. A total of 30.8% of the participants who used the free text field stated that they also obtained information from their doctors, and 26.6% reported that they also sought information through personal conversations with friends and acquaintances.

**Figure 1:**
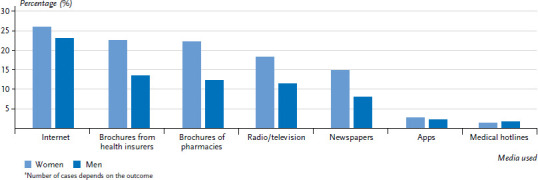
Percentage of participants who ‘often’ use media when searching for health information by sex, age and socioeconomic status (n=2,859 women; n=2,187 men)^*^ Source: KomPaS study (2017)

Figure 1 depicts the search for health information by women and men in order of the type of media that they ‘often’ use.

Figure 1 demonstrates that women who ‘often’ use media to search for health information use all types of media more frequently than men do, with the exception of medical hotlines provided by health insurers. The sex-based differences that are clear from Figure 1 are statistically significant (apart from differences in the use of apps and medical hotlines provided by health insurers). The Internet is the most popular choice for both women (26.0%) and men (23.1%) out of all of the listed types of media in the ‘often’ category. Other studies have observed the same sex-based results [13–16]. Baumann et al. (2017) extensively address sex-specific determinants and patterns of online behaviour with regard to the search for health information [13]. Marstedt (2018) discusses the general motives for searching for health information on the Internet [10]. The results of the analyses undertaken by the KomPaS study are consistent with those of various others in terms of usage behaviour by different age groups [13–16].

Table 1 presents the respondents who ‘often’ use the Internet as a source of health information by age and sex. Women aged 65 or below use the Internet to search for health information more frequently than men in the same age group. This difference is statistically significant and particularly prominent among the 30 to 44 age group. Women are presumably more closely involved with issues related to health and illness and also more likely to act as a family’s primary health informant than men are [2, 17].

In the group aged 65 and over, however, the statistically significant sex-based differences are reversed. It will be interesting to see whether this changes in the future.

Socioeconomic status also has a major impact on health information-seeking behaviour. Previous studies have shown that people in the low socioeconomic status group search for health information and use e-health services less often [5, 14–15, 18–19]. These results are confirmed by the KomPaS study. The sex-based differences in the search for health information on the Internet are also evident within socioeconomic status groups, with significant differences between the sexes in the low and medium status groups. Women in the low socioeconomic status group who ‘often’ use the Internet to search for health information do so less frequently than their male counterparts in the same status group. This sex-based relationship is reversed in the medium socioeconomic status group. At the same time, women in the high socioeconomic status group who ‘often’ use the Internet to search for health information do so more frequently than their male counterparts in the same status group. However, this difference is not statistically significant.

The results from the KomPaS study show that the search for health information on the Internet follows population group-specific patterns. These differences need to take into account when attempts are made to improve people’s capacity to make decisions and to bolster their health literacy through the provision of Internet-based health information.

## Key statements

- Data from the KomPaS study demonstrate a high level of interest in health-related topics.
- The Internet is the first choice among women and men who use media to search for health information.
- Up to the age of 65, women use the Internet to search for health information more frequently than men do. This is reversed from the age of 65.
- People in the low socioeconomic status group search the Internet for health information less often than people in the high or medium status groups.

## Figures and Tables

**Table 1:** Percentage of participants who ‘often’ use the Internet when searching for health information by sex, age and socioeconomic status (n=2,859 women; n=2,187 men) Source: KomPaS study (2017)

	%	(95% CI)
**Women (total)**	**26.0**	**(23.8-28.4)**
**Age group** 18-29 years 30-44 years 45-64 years ≥65 years	36.7 46.4 25.5 4.8	(28.6-45.6) (40.2-52.7) (22.6-28.6) (3.7-6.1)
**Socioeconomic status** Low Medium High	11.4 26.7 36.6	(6.2-20.1) (23.8-29.7) (32.5-40.9)
**Men (total)**	**23.1**	**(20.8-25.6)**
**Age group** 18–29 years 30–44 years 45–64 years ≥65 years	35.4 27.1 21.9 11.3	(28.1–43.5) (21.4–33.7) (18.8–25.3) (9.1–13.9)
**Socioeconomic status** Low Medium High	19.2 20.5 30.3	(12.7–28.0) (17.4–23.9) (26.6–34.2)
**Total (women and men)**	**24.6**	**(23.0-26.3)**

CI = confidence interval
